# The American and Colonial Journals: Surgery

**Published:** 1838-10

**Authors:** 


### SURGERY.

### *Operations for the Excision of the Ribs.* By Dr. Warren, of Boston.

Case i. The patient was a gentleman, forty years of age, a patient of Dr. Bigelow. In the early part of his life he was subject to severe attacks of asthma; this he has been free from the last twelve years, during which time he has enjoyed good health, until the summer of 1834, when he had the typhus fever. On his recovery, after an illness of six weeks, he was troubled with a severe pain in the right side, in the region of the angle of the sixth and seventh ribs. A small tumour shortly made its appearance at this point, which, after having remained stationary for some months, finally opened and discharged a quantity of sanious pus. In the spring of 1835, Dr. Warren was consulted on account of the fistulous cavity which had remained since the opening of the abscess. Upon examination, a small aperture was discovered on the side of the chest, large enough to admit a common-sized probe, and situated over the point where the sixth and seventh ribs are joined to their cartilages. Upon passing a probe into this aperture, it penetrated rather more than an inch in a direction downwards, and backwards, where it was resisted by a firm, but not osseous substance. This examination gave great pain. During the following winter, the pains about the ribs increased, the respiration began to be more or less affected, and the patient was occasionally troubled with hiccough.

Finding himself prevented from attending to his business, gradually losing strength, and, added to this, much suffering attending the disease, he finally determined to submit himself to an operation. The patient was placed on a table, in a horizontal position. An incision four inches in length, was made in an oblique direction, over the sixth rib, joined by two incisions at either extremity of the first, so as by this means to form two quadrangular flaps. The integuments were now dissected up, a matter of some difficulty, from their very strong adhesions to the parts below, and a firm cartilaginous substance exposed, forming a large tumour, destroying the natural appearance of the parts, and rendering it difficult to determine in which direction the diseased ribs and cartilages lay. After much time and patience, this substance was carefully removed, partly by shaving it off with the scalpel, and by occasionally removing portions of it with the cutting forceps, and the diseased parts at length exposed. Both the sixth and seventh ribs were found to be carious, their cartilages also being involved. The ribs were now carefully isolated from the surrounding parts, a probe passed under them, and the pleura, which was much thickened, and a part of the diaphragm, separated from their internal surfaces. Three inches of the seventh rib, and its cartilage, with two inches of the sixth, were removed by the chain-saw and cutting forceps. There was very little hemorrhage, and no appearance of bleeding from the intercostal arteries, which undoubtedly had become obliterated in the course of the disease. The external wound, which had been somewhat enlarged in the course of the operation, was brought together by two or three points of suture, and by adhesive straps. Although the operation lasted a long time, and some parts of it were exceedingly painful, it was borne with great fortitude. The patient did not seem greatly exhausted.

On the second day after the operation, he had a severe pleuritic attack. This yielded to a copious bleeding, and in the course of two weeks he was able to walk about his chamber. From exerting himself too much, he was attacked with an erythematous affection between the wound and os ilii. This terminated in a partial suppuration. From the consequences of this attack he very slowly recovered. The following summer he went into the country, still suffering occasionally with some pain about the ribs. During his residence in the country, his health gradually improved; and on his return to town, the wound had entirely healed, and he is now in the full enjoyment of health.

One of the greatest difficulties in the operation was the removal of the cartilaginous formation, which covered the ribs, and destroyed all the anatomical signs which were necessary for prosecuting the operation with anything like facility. This occupied much time, and the parts, contrary to what would naturally be supposed, were endowed with an excessive degree of sensibility. A portion of the diaphragm and pleura, was exposed by the operation. The latter, during the slow inflammation which had been going on, had become greatly thickened; if this had not been the case, it would have been very difficult to have separated the pleura, without cutting into the cavity of the chest.

CASE II. The patient was a stout, healthy person, a surveyor, from Upper Canada. Six years since, and without any previous injury that he was aware of, a small hard tumour appeared over the angle of the ninth or tenth rib. From that time to the present, it has gradually increased in size—having, however, daily less of that bony hardness, which characterized it at first.

It was of a circular form, about six inches in diameter, and having an elevation above the ribs of between two and three inches. Its situation was on the lower part of the chest, covering a portion of the seventh, eighth, ninth, and tenth ribs, to all of which it appeared attached, but more firmly to the ninth, which was most affected by its movements. The skin covering the tumour was perfectly natural; both in colour and consistence, and the patient complained of no inconvenience or suffering from the disease.

The operation was performed on the 27th of March, 1837, in the following manner. The patient was placed on a table a little inclined to the right, with a pillow under the chest, so as to cause a projection of the left side of the thorax. The operation was commenced by a longitudinal incision about five inches in length, directly over the tumour. This was joined by another transverse incision at right angles with its central part—the two incisions being well represented by the letter T. The flap thus formed being dissected up, the insertions of the external oblique were exposed and dissected off, not without some difficulty, however, on account of its strong contractions. The latissimus dorsi was now discovered passing over the outer edge of the tumour, and the same, and in fact more trouble was experienced in dividing this muscle than the preceding; the dissection of it was excessively painful to the patient, and was followed by some hemorrhage. The tumour being at length perfectly exposed was found to originate from the ninth rib, but was strongly adherent to the seventh, eighth, and tenth. A knife was now carefully insinuated under the tumour, and its adhesions to the ribs dissected off, great care being taken not to cut through the intercostal muscle and penetrate the chest. As the attachment of the tumour to the ninth rib was not by its whole base, it was thought that more of the rib might be saved by first detaching the tumour and afterwards cutting out the rib, than by removing both the tumour and rib together. It was therefore cut off from the rib at about an inch distance from its cartilage, and the morbid origin of the tumour exposed. The intercostals were now cut through, the diaphragm carefully separated from the rib and pleura, and a director passed under at the points where the rib was to be divided. The bone was next cut through with the cutting forceps, and about two inches in length of it removed, with a portion of its cartilage. The diaphragm immediately rose up, forming a hernia between the ribs. The hemorrhage was not great—most of it being from the divided muscles. No artery bled sufficiently to require being secured by ligature. The wound was brought together by sutures, and adhesive plasters. The patient was very little exhausted by the operation. A considerable degree of febrile excitement followed, requiring the employment of copious bleeding. The wound has united nearly throughout by the first intention, and the patient is rapidly gaining strength, without the occurrence of any bad symptoms.

In this case, unlike the preceding, very little thickening of the parts lying under the ribs had taken place. This rendered the difficulty and danger much greater in separating the pleura and diaphragm from their adhesions to the ribs, which, however, was finally accomplished without penetrating the chest.

*Boston Medical and Surgical Journal. May* 3, 1837.

---

### *Two Cases of Double Amputation of the Legs, from Frost-Bite.*

By J. A. Sewell, m.d., Surgeon to the Hôtel Dieu, Quebec.

I. James Brown, æt. twenty-seven, was admitted into the Hôtel Dieu in May, 1833, under the care of Dr. Morrin. He was a sailor, and had been shipwrecked the previous autumn in the Gulf of St. Lawrence, and was exposed for a considerable time to intense cold by which both legs were frozen: he got with difficulty to the nearest habitation, where he remained all the winter without medical assistance, enduring extreme privations and sufferings. On admission, both limbs were found to be in a complete state of sphacelation, the lower ends of the tibiæ and fibulæ, together with the bones and ligaments of the ankle joints being entirely exposed, and his whole general appearance indicating great constitutional disturbance. It was at once apparent that amputation alone could save life. The question, however, whether one limb should be removed, and some time allowed for the system to rally before the second operation should be undertaken, or, on the other hand, whether the double amputation should be simultaneously performed, could not so readily be decided. After due deliberation it was determined that the latter plan should be adopted, and the two operations were accordingly performed at the same moment, by Dr. Morrin and the late Dr. Hall. The operations occupied about the usual time, and the man recovered without one untoward symptom, and was discharged perfectly cured within the month.

II. A young Canadian came under Dr. James A. Sewell's care in March last, in the Hôtel Dieu. He was of very intemperate habits, and one night in January, when intoxicated, lost his way on the ice between Quebec and the island of Orleans. He was found the next morning with both feet completely frozen. When Dr. S. first saw him, mortification had extended about midway up each leg, the bones of which were, to a considerable extent, perfectly denuded, the feet remaining attached by the lateral ligaments alone. He was at this time losing ground rapidly; the appetite entirely gone, with profuse night sweats and colliquative diarrhœa; he expressed an earnest desire for the immediate removal of the legs. Double amputation, as in the foregoing case, was accordingly performed by Drs. Sewell and Perrant. The first dressings were removed on the fifth day, when both stumps were found almost completely united by the first intention. The man recovered rapidly and perfectly, and was discharged in about five weeks from the day of the operation.

[Whether it is advisable to perform double amputation in cases requiring it, simultaneously or successively, is a question to be decided by experience alone: these cases, as far as they go, are valuable data towards forming an opinion, and they do much credit to the surgical skill and intrepidity of Dr. Sewell and his coadjutors. It should be remembered the patients although reduced were both young men.]

*The Boston Medical and Surgical Journal. November* 15, 1837.

